# HoxB13 expression in ductal type adenocarcinoma of prostate: clinicopathologic characteristics and its utility as potential diagnostic marker

**DOI:** 10.1038/s41598-019-56657-8

**Published:** 2019-12-27

**Authors:** Cheol Keun Park, Su-Jin Shin, Yoon Ah Cho, Jin Woo Joo, Nam Hoon Cho

**Affiliations:** 1 Department of Pathology, Severance Hospital, Yonsei University College of Medicine, Seoul, Republic of Korea; 2 Department of Pathology, Armed Forces Capital Hospital, Seongnam, Republic of Korea; 3 Department of Pathology, Hanyang University College of Medicine, Seoul, Republic of Korea; 4 Department of Pathology and Translational genomics, Samsung medical center, Seoul, Republic of Korea

**Keywords:** Diagnostic markers, Surgical oncology, Prostate

## Abstract

The histologic criteria and selective biomarkers of prostate ductal type adenocarcinoma (DAC) are relatively unknown compared to that known about acinar type adenocarcinoma (AAC). It is known that genetic alteration in *Hox13* gene is associated with carcinogenesis of prostate cancer. In this study, we investigated clinicopathologic characteristics of HoxB13 expression in prostate cancer and compared clinicopathologic profiles of DAC and AAC of prostate. After slide review, some morphological variants of DAC, equivalent to Gleason pattern 3 and 5 of AAC were identified. High level of HoxB13 expression was identified in 46.5% (46 out of 99 cases) and 39.2% (31 out of 79 cases) of cases that belong to the training set and test set, respectively. In the training set, high level of HoxB13 expression was significantly correlated with DAC (*P* < 0.001), higher Gleason score (*P* < 0.001), advanced pathologic T stage (*P* = 0.010), and occurrence of biochemical recurrence (BCR; *P* < 0.001). The test set confirmed that high level of HoxB13 expression was associated with DAC (*P* < 0.001), higher Gleason score (*P* = 0.001), advanced pathologic T stage (*P* < 0.001), and occurrence of BCR (*P* < 0.001). Our findings suggest that HoxB13 may be a useful diagnostic marker for detection of DAC and a prognostic marker for prediction of BCR.

## Introduction

Prostate cancer is one of the most common cancers in males, especially in developed countries^1^. The majority of prostate cancer is acinar type adenocarcinoma (AAC); however, there are several variants of prostate cancer causing diagnostic difficulties due to the overlapping features with AAC^2^. Thus, variant forms are often misdiagnosed as AAC when using histology samples, causing difficulties in the histologic evaluation of prostate cancer^3^.

Ductal type adenocarcinoma (DAC) is another common subtype of prostate adenocarcinoma, and its incidence has been gradually increasing^4,5^. When compared with AAC, patients with DAC are more often diagnosed with an advanced T stage and exhibit greater mortality^4,6^. Generally, DAC shows papillary architecture lined by pseudostratified columnar epithelium with voluminous cytoplasm^6,7^; however, due to its broad spectrum of morphological presentations, DAC cases are often assigned one of several differential diagnoses: metastatic adenocarcinoma from the colorectal area, urothelial carcinoma, high-grade prostatic intraepithelial neoplasia (HGPIN), and intraductal prostate cancer (IDC-P)^7,8^.

A study about interobserver variabilities in the diagnosis of DAC was conducted, and among several diagnostic parameters, papillary architecture was found to be the most useful feature for diagnosis of DAC. Interobserver discrepancies, however, still remain a major obstacle in its diagnosis^3^. To address this problem, several studies have been performed to identify diagnostic markers of DAC; although, distinguishing DAC from AAC remains difficult^9–11^.

*Hox* genes, composed of four paralogous clusters, are located on four different chromosomes^12^. Among these genes, posterior *Hox* genes, particularly *HoxA13*, *HoxB13*, and *HoxD13*, are important for the development of the separate lobes of the prostate gland, seminal vesicles, and epididymis. In addition, each *Hox13* gene is associated with a lobe-specific prostatic identity and cellular differentiation^13,14^. Genetic alteration of *HoxA13* and *HoxB13* genes is associated with the development of prostate cancer^14–17^. Specifically, a germline G84E mutation in *HoxB13* was associated with hereditary prostate cancer^16^. Furthermore, dysregulation of *HoxB13* has been reported in colon, breast, and lung cancers as well as in cutaneous melanoma^18–20^. Despite these findings, differential HoxB13 expression according to histologic subtype and the clinical implications of Hox expression in prostate cancer have not been fully investigated. Thus, in this study, we evaluated the expression of HoxA13 and HoxB13 in DAC versus AAC to identify their roles as diagnostic markers for DAC.

## Results

### Histopathologic reassessment of 178 prostate cancer cases

After the slide review, 25 cases previously diagnosed as DAC, including 19 equivocal cases, were reclassified as AAC, and 18 cases previously diagnosed as AAC were reclassified as DAC. Therefore, 68 cases of DAC and 110 cases of AAC were used for comparison of the clinicopathologic characteristics based on the histologic subtypes.

**Figure 1:**
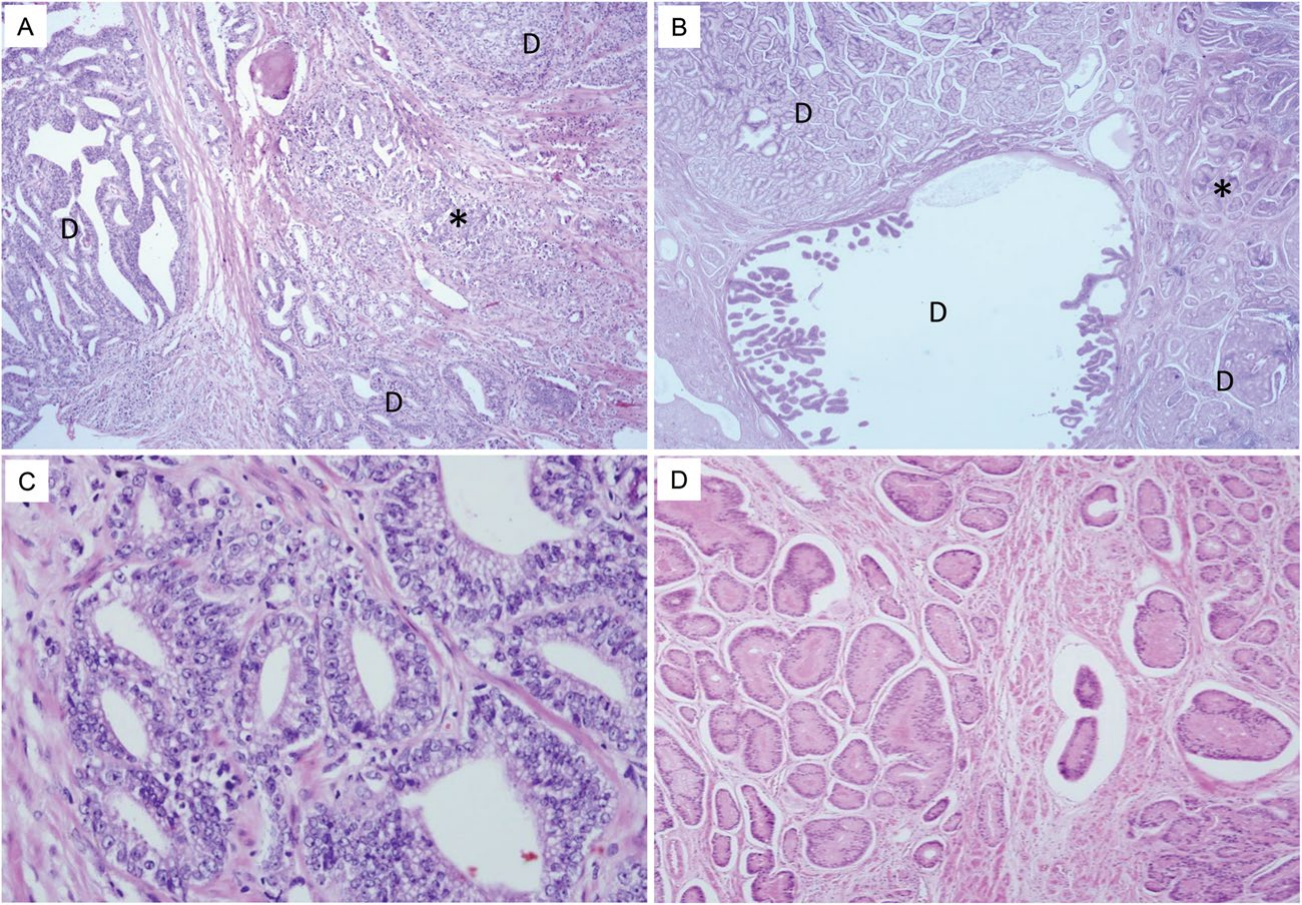
Morphological features of representative cases of Gleason pattern 3 DAC. (**A**,**B**) The majority of DACs shows Gleason pattern 4 and arose from the inner zone of periurethral primary ducts with an expansive invasive pattern (marked as “D”). Some tubular structures which is adjacent to Gleason pattern 4 DACs are identified (marked with asterisk). (**C**,**D**) On the higher magnification of areas marked with asterisk, these tubular structures are composed of tall columnar tumor cells with elongated nuclei.

DACs that fulfilled all diagnostic criteria for ductal component were all assigned as Gleason pattern 4. The majority of cases arose from the inner zone of periurethral primary ducts with expansile invasive pattern (Fig. 1A,B). They showed complex papillary patterns with fibrovascular cores, and the papillary architecture was composed of tall columnar tumor cells, mimicking colon or endometrioid adenocarcinoma. Coagulative necrosis was rarely identified, unlike in colon or endometrioid type adenocarcinoma. In addition, less pleomorphism and stratification were identified compared to those of urothelial carcinoma.

In addition to the Gleason pattern 4 DACs, which fulfilled all diagnostic criteria, 10 cases that had been originally classified as DAC showed some morphological variants in the juxtaposed: 6 cases with tubular structures, 2 cases with growth patterns equivalent to Gleason pattern 5 of AAC and 2 cases with both variants. Simple tubular structures were composed of tall columnar tumor cells with elongated nuclei and were intermingled with conventional DACs; however, no evidence of papillary cores was observed (Fig. 1C,D). These tubular structures showed different morphological features than those of AAC, raising the possibility of DACs with Gleason pattern 3.

**Figure 2:**
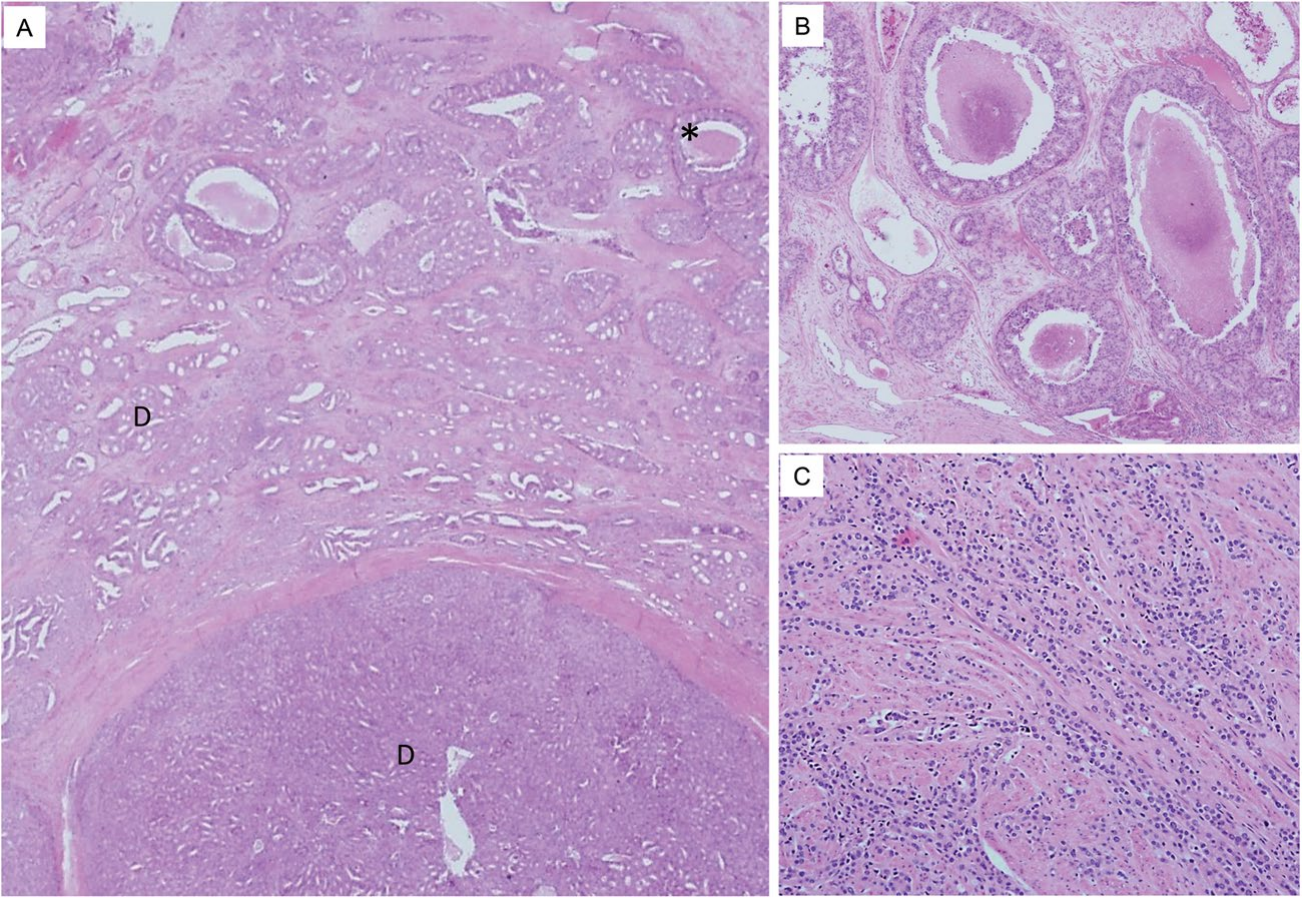
Morphological features of representative cases of Gleason pattern 5 DAC. (**A**) Gleason pattern 4 DACs (marked as “D”) shows expansile invasive pattern and some morphological variants are identified at the periphery of Gleason pattern 4 DACs (marked with asterisk). (**B**) On the higher magnification of areas marked with asterisk, tumor cell nests with central comedo-type necrosis are observed adjacent to the Gleason pattern 4 DACs. Comparatively larger in size than Gleason pattern 5 of AAC. (**C**) In other cases, infiltrative cord-like patterns mimic invasive lobular carcinoma are noted.

Two growth patterns equivalent to Gleason pattern 5 of AAC were observed adjacent to conventional DACs (Fig. 2A). Two cases showed central comedo-type necrosis (Fig. 2B). Having a comparatively larger size than AAC was a prerequisite for determination of DAC. Infiltrative cord-like patterns that mimicked invasive lobular carcinoma of the breast was observed in two cases (Fig. 2C).

### Clinicopathologic features of patients

Clinicopathologic analysis was performed on the training set and test set, respectively. In the training set, DAC was significantly associated with a higher Gleason score (*P* < 0.001), tumor volume more than 5cc (*P* = 0.004), presence of extraprostatic extension (EPE; *P* = 0.004), advanced pathologic T stage (*P* = 0.016), intact PTEN expression (*P* < 0.001) and occurrence of biochemical recurrence (BCR; *P* = 0.004). The results of clinicopathologic analysis on the test set was similar to that of training set. DAC was significantly correlated with a higher Gleason score (*P* < 0.001), presence of EPE (*P* < 0.001), advanced pathologic T stage (*P* < 0.001), ERG positivity (*P* = 0.012) and occurrence of BCR (*P* = 0.004). The results of chi-square analysis of the clinicopathologic factors are summarized in Table 1.

**Table 1:** Clinicopathological characteristics of 178 prostate cancers according to the histologic subtype in training set and test set.

Category	Variables	Training set						Test set					
		No. of cases (*n* = 99)	DAC (%) (*n* = 36)		AAC (%) (*n* = 63)		*P-*value	No. of cases (*n* = 79)	DAC (%) (*n* = 32)		AAC (%) (*n* = 47)		*P-*value
Age (y)			67.7 ± 7.90		66.0 ± 7.59		0.272		66.3 ± 5.90		65.2 ± 7.79		0.513
Pre-operative PSA (ng/mL)			12.8 ± 9.76		11.4 ± 14.5		0.612		11.7 ± 7.46		9.54 ± 6.99		0.179
Gleason score	8	69	17	(47.2)	52	(82.5)	<0.001	55	15	(46.9)	40	(85.1)	<0.001
	9–10	30	19	(52.8)	11	(17.5)		24	17	(53.1)	7	(14.9)	
Location	Unilateral	23	8	(22.2)	15	(23.8)	0.857	14	5	(15.6)	9	(19.1)	0.687
	Bilateral	76	28	(77.8)	48	(76.2)		65	27	(84.4)	38	(80.9)	
Tumor volume	≤5 cc	74	21	(58.3)	53	(84.1)	0.004	60	21	(65.6)	39	(83.0)	0.076
	>5 cc	25	15	(41.7)	10	(15.9)		19	11	(34.4)	8	(17.0)	
EPE	Absent	52	12	(38.9)	40	(31.7)	0.004	47	10	(31.3)	37	(78.7)	<0.001
	Present	47	24	(61.1)	23	(68.3)		32	22	(68.7)	10	(21.3)	
PNI	Absent	7	3	(8.3)	4	(6.3)	0.703	5	3	(9.4)	2	(4.3)	0.390
	Present	92	33	(91.7)	59	(93.7)		74	29	(90.6)	45	(95.7)	
LVI	Absent	88	31	(86.1)	57	(90.5)	0.522	67	25	(78.1)	42	(89.4)	0.210
	Present	11	5	(13.9)	6	(9.5)		12	7	(21.9)	5	(10.6)	
RM extension	Absent	34	14	(43.4)	20	(41.7)	0.472	42	18	(56.3)	24	(51.1)	0.650
	Present	65	22	(58.6)	43	(58.3)		37	14	(43.7)	23	(48.9)	
SV involvement	Absent	82	27	(75.0)	55	(87.3)	0.118	69	25	(78.1)	44	(93.6)	0.081
	Present	17	9	(25.0)	8	(12.7)		10	7	(21.9)	3	(6.4)	
Pathologic T stage	T2	46	11	(30.6)	35	(55.6)	0.016	47	10	(31.3)	37	(78.7)	<0.001
	T3 and T4	53	25	(69.4)	28	(44.4)		32	22	(68.7)	10	(21.3)	
Pathologic N stage*	N0	68	32	(88.9)	36	(100.0)	0.115	40	26	(96.3)	14	(100.0)	>0.999
	N1	4	4	(11.1)				1	1	(3.7)			
PTEN IHC	Intact	55	30	(83.3)	25	(39.7)	<0.001	33	10	(31.3)	23	(48.9)	0.118
	Loss	44	6	(16.7)	38	(60.3)		46	22	(68.7)	24	(51.1)	
ERG IHC	Negative	83	32	(88.9)	51	(81.0)	0.302	60	29	(90.6)	31	(66.0)	0.012
	Positive	16	4	(11.1)	12	(19.0)		19	3	(9.4)	16	(34.0)	
BCR	Absent	57	14	(38.9)	43	(68.3)	0.004	61	19	(59.4)	42	(89.4)	0.004
	Present	42	22	(61.1)	20	(31.7)		18	13	(40.6)	5	(10.6)	

Abbreviations: DAC, ductal type adenocarcinoma; AAC, acinar type adenocarcinoma; PSA, prostate-specific antigen; EPE, extraprostatic extension; PNI, perineural invasion; LVI, lymphovascular invasion; RM, resection margin; SV, seminal vesicle; IHC, immunohistochemistry; BCR, biochemical recurrence.

*Evaluated in 113 prostatectomy specimens.

### Clinicopathologic characteristics according to HoxA13 and HoxB13 expression status

**Figure 3:**
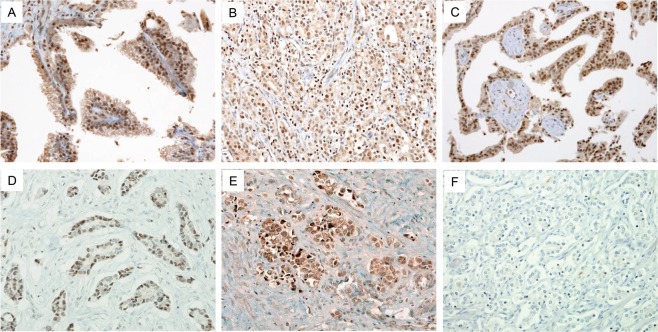
Expression of HoxA13 and HoxB13 in prostate cancer. HoxA13 and HoxB13 is expressed in the nuclei with non-specific cytoplasmic staining. (**A**) In Gleason pattern 4 DACs, HoxA13 is expressed in tumor cells, especially those surrounding papillary cores and comprising large ducts. (**B**) In morphological variants of DAC and AAC, HoxA13 expression is weaker compared to that of Gleason pattern 4 DACs. (**C**) Similar to HoxA13, HoxB13 is expressed in tumor cells of Gleason pattern 4 DACs, with especially strong expression in cells surrounding papillary cores and comprising large ducts. (**D**) DACs with a Gleason pattern 3 also showed high level of HoxB13 expression. (**E**) High level of HoxB13 expression is also identified in DACs with a Gleason pattern 5. (**F**) All of the AACs showed low level of HoxB13 expression except for 19 equivocal cases.

In the almost all of cases, HoxA13 and HoxB13 were expressed in the nucleus of tumor cells, with concomitant non-specific cytoplasmic staining. HoxA13 is highly expressed in tumor cells of Gleason pattern 4 DACs, especially those surrounding papillary cores and comprising large ducts (Fig. 3A). In morphological variants of DAC and AAC, the expression of HoxA13 is relatively weaker compared to that of Gleason pattern 4 DACs (Fig. 3B). For HoxB13, the expression patterns within each tumor subtype and Gleason pattern differed. The expression of HoxB13 was similar to that of HoxA13 in Gleason pattern 4 DACs (Fig. 3C). Morphological variants of DAC, which is located adjacent to Gleason pattern 4 DACs, also showed expression of HoxB13 in columnar cells of Gleason pattern 3 DACs (Fig. 3D) and singly scattered tumor cells or those comprising large nests with central comedo-type necrosis of Gleason pattern 5 DACs (Fig. 3E). On the contrary, HoxB13 expression was lower in AAC cases compared with DACs (Fig. 3F). All of 19 equivocal cases showed high HoxB13 expression. The validation results of HoxA13 and HoxB13 antibody are presented in Supplementary Fig. 1.

High HoxB13 expression was identified in 46.5% (46 out of 99 cases) and 39.2% (31 out of 79 cases) of cases that belong to the training set and test set, respectively. In the training set, high level of HoxB13 expression was significantly associated with DAC (*P* < 0.001), a higher Gleason score (*P* < 0.001), tumor volume more than 5cc (*P* = 0.042), presence of EPE (*P* = 0.001), advanced pathologic T stage (*P* = 0.010), intact PTEN expression (*P* < 0.001) and occurrence of BCR (*P* < 0.001). The test set showed the results similar to those of the training set. High level of HoxB13 expression was significantly correlated with DAC (*P* < 0.001), a higher Gleason score (*P* = 0.001), presence of EPE (*P* < 0.001), presence of lymphovascular invasion (LVI; *P* = 0.001), involvement of seminal vesicle (*P* = 0.043), advanced pathologic T stage (*P* < 0.001), lower ERG expression (*P* = 0.016), and occurrence of BCR (*P* < 0.001). In addition, high level of HoxB13 expression showed a tendency toward tumor volume more than 5cc (*P* = 0.056), and frequent intact PTEN expression (*P* = 0.065). The results of chi-square analysis are summarized in Table 2.

**Table 2:** Clinicopathological characteristics of 178 prostate cancers HoxB13 expression status in training set and test set.

Category	Variables	Training set						Test set					
		No. of cases (*n* = 99)	High (%) (*n* = 46)		Low (%) (*n* = 53)		*P-*value	No. of cases (*n* = 79)	High (%) (*n* = 31)		Low (%) (*n* = 48)		*P-*value
Age (y)			68.2 ± 7.80		65.3 ± 7.45		0.062		65.9 ± 6.66		65.6 ± 7.38		0.871
Pre-operative PSA (ng/mL)			11.8 ± 9.05		12.0 ± 15.7		0.934		11.1 ± 7.81		10.0 ± 6.87		0.516
Histologic subtype	AAC	63	13	(28.3)	50	(94.3)	<0.001	47	6	(19.4)	41	(85.4)	<0.001
	DAC	36	33	(71.7)	3	(5.7)		32	25	(80.6)	7	(14.6)	
Gleason score	8	69	23	(50.0)	46	(86.8)	<0.001	55	15	(48.4)	40	(83.3)	0.001
	9–10	30	23	(50.0)	7	(13.2)		24	16	(51.6)	8	(16.7)	
Location	Unilateral	23	8	(17.4)	15	(28.3)	0.200	14	7	(22.6)	7	(14.6)	0.363
	Bilateral	76	38	(82.6)	38	(71.7)		65	24	(77.4)	41	(85.4)	
Tumor volume	≤ 5 cc	74	30	(65.2)	44	(83.0)	0.042	60	20	(64.5)	40	(83.3)	0.056
	> 5 cc	25	16	(34.8)	9	(17.0)		19	11	(35.5)	8	(16.7)	
EPE	Absent	52	16	(34.8)	36	(67.9)	0.001	47	10	(32.3)	37	(77.1)	<0.001
	Present	47	30	(65.2)	17	(32.1)		32	21	(67.7)	11	(22.9)	
PNI	Absent	7	4	(8.7)	3	(5.7)	0.701	5	3	(9.7)	2	(4.2)	0.376
	Present	92	42	(91.3)	50	(94.3)		74	28	(90.3)	46	(95.8)	
LVI	Absent	88	40	(87.0)	48	(90.6)	0.569	67	21	(67.7)	46	(95.8)	0.001
	Present	11	6	(13.0)	5	(9.4)		12	10	(32.3)	2	(4.2)	
RM extension	Absent	34	16	(34.8)	18	(34.0)	0.932	42	17	(54.8)	25	(52.1)	0.811
	Present	65	30	(65.2)	35	(66.0)		37	14	(45.2)	23	(47.9)	
SV involvement	Absent	82	35	(76.1)	47	(88.7)	0.098	69	24	(77.4)	45	(93.8)	0.043
	Present	17	11	(23.9)	6	(11.3)		10	7	(22.6)	3	(6.2)	
Pathologic T stage	T2	46	15	(32.6)	31	(58.5)	0.010	47	10	(32.3)	37	(77.1)	<0.001
	T3 and T4	53	31	(67.4)	22	(41.5)		32	21	(67.7)	11	(22.9)	
Pathologic N stage*	N0	68	40	(90.9)	28	(100.0)	0.152	40	23	(100.0)	17	(94.4)	0.439
	N1	4	4	(9.1)				1			1	(5.6)	
PTEN IHC	Intact	55	36	(78.3)	19	(35.8)	<0.001	46	22	(71.0)	24	(50.0)	0.065
	Loss	44	10	(21.7)	34	(64.2)		33	9	(29.0)	24	(50.0)	
ERG IHC	Negative	83	40	(87.0)	43	(81.1)	0.432	60	28	(90.3)	32	(66.7)	0.016
	Positive	16	6	(13.0)	10	(18.9)		19	3	(9.7)	16	(33.3)	
BCR	Absent	57	15	(32.6)	42	(79.2)	<0.001	61	15	(48.4)	46	(95.8)	<0.001
	Present	42	31	(67.4)	11	(20.8)		18	16	(51.6)	2	(4.2)	

Abbreviations: DAC, ductal type adenocarcinoma; AAC, acinar type adenocarcinoma; PSA, prostate-specific antigen; EPE, extraprostatic extension; PNI, perineural invasion; LVI, lymphovascular invasion; RM, resection margin; SV, seminal vesicle; IHC, immunohistochemistry; BCR, biochemical recurrence

*Evaluated in 113 prostatectomy specimens.

High HoxA13 expression was identified in 60.6% (60 out of 99 cases) and 58.2% (46 out of 79 cases) of cases that belong to the training set and test set, respectively. In the training set, high level of HoxA13 expression was significantly associated with presence of EPE (*P* = 0.008), and advanced pathologic T stage (*P* = 0.015). However, no significant correlation between high HoxA13 expression and various clinicopathologic factors was identified in the test set. The results of chi-square analysis are summarized in Supplementary Table S1, and representative immunoprofiles are presented in Supplementary Fig. 2.

### Interobserver agreement

Prior to estimating the interobserver agreement, we established two diagnostic criteria for the reproducible assessment of DAC. *Morphologic criteria* were described in the Materials and methods section as a diagnostic criteria for the ductal component. *Immunophenotypic criteria* were based on the *morphologic criteria* and HoxB13 expression. Equivocal cases that exhibited high level of HoxB13 expression were classified as DAC.

In the training set, interobserver agreement was 66.7% (63 out of 99 cases), and kappa value was 0.214 [95% confidence interval (CI), 0.017–0.418]) without specific diagnostic criteria. However, in the second round, following application of the *morphologic criteria*, the interobserver agreement increased to 69.7% (69 out of 99 cases), and the kappa value was 0.353 (95% CI, 0.154–0.539). With the additional application of the *immunophenotypic criteria*, the interobserver agreement further increased to 83.8% (83 out of 99 cases), and the kappa value was 0.670 (95% CI, 0.515–0.805). In the test set, the interobserver agreement after the application of the *morphologic criteria* was 75.9% (60 out of 79 cases) and kappa value was 0.498 (95% CI, 0.286–0.677). With application of the *immunophenotypic criteria*, the interobserver agreement further increased to 83.5% (66 out of 79 cases), and the kappa value was 0.657 (95% CI, 0.476–0.821).

### Impact of histologic subtype and HoxB13 expression on BCR-free survival and prognosis

**Figure 4:**
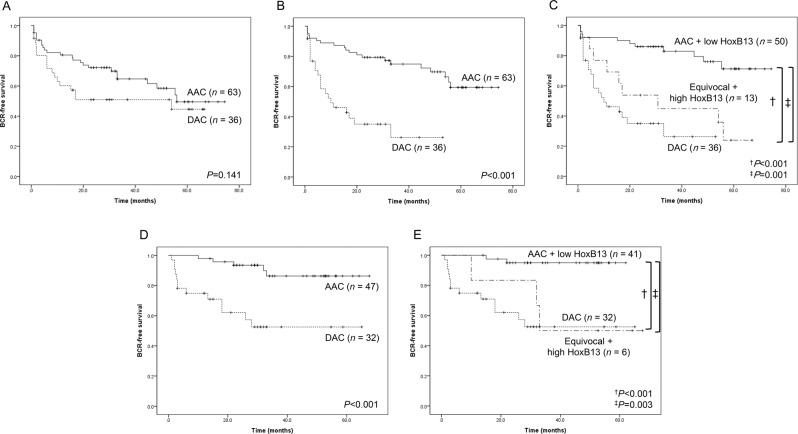
BCR-free survival of 178 prostate cancer patients. (**A**) In the training set, no significant differences in BCR-free survival between AAC and DAC were observed (*P* = 0.141). (**B**) When applying *morphologic criteria*, DAC and AAC were found to have significantly different BCR-free survival (*P* < 0.001). (**C**) When applying *immunophenotypic criteria*, DAC cases showed significantly shorter BCR-free survival than AAC cases with low level of HoxB13 expression, and 13 equivocal cases with high level of HoxB13 expression showed similar BCR-free survival as DAC cases (*P* < 0.001 and *P* = 0.001, respectively). (**D**) When applying *morphologic criteria* in the test set, DAC and AAC showed significantly different BCR-free survival (*P* < 0.001). (**E**) When applying *immunophenotypic criteria* in the test set, DAC cases had significantly shorter BCR-free survival than AAC cases with low level of HoxB13 expression, and 6 equivocal cases with high level of HoxB13 expression showed similar BCR-free survival as DAC cases (*P* < 0.001 and *P* = 0.003, respectively). ^†^Refers to the comparison between AACs with low HoxB13 expression and DACs. ^‡^Refers to the comparison between AACs with low HoxB13 expression and equivocal cases with high HoxB13 expression.

In the training set, no significant differences in BCR-free survival between AAC and DAC were observed (*P* = 0.141; Fig. 4A) before the application of our diagnostic criteria. However, after the application of our diagnostic criteria, significant difference was identified between the subgroups. When applying *morphologic criteria*, DAC and AAC were found to have significantly different BCR-free survival (*P* < 0.001; Fig. 4B). When based on *immunophenotypic criteria*, DAC cases had significantly shorter BCR-free survival than AAC cases with low level of HoxB13 expression, and 13 equivocal cases with high level of HoxB13 expression showed similar BCR-free survival as DAC cases (*P* < 0.001 and *P* = 0.001, respectively; Fig. 4C).

Survival analysis on the test set validated our diagnostic criteria for DAC. When applying *morphologic criteria* in the test set, DAC and AAC showed significantly different BCR-free survival (*P* < 0.001; Fig. 4D). When applying *immunophenotypic criteria*, DAC cases had significantly shorter BCR-free survival than AAC cases with low level of HoxB13 expression, and 6 equivocal cases with high level of HoxB13 expression showed similar BCR-free survival as DAC cases (*P* < 0.001 and *P* = 0.003, respectively; Fig. 4E).

Univariate analysis identified the following characteristics that were associated with shorter BCR-free survival: DAC (*P* < 0.001), higher Gleason score (*P* < 0.001), bilateral location (*P* = 0.032), tumor volume more than 5 cc (*P* < 0.001), presence of EPE (*P* < 0.001), presence of LVI (*P* = 0.001), extension to resection margin (*P* < 0.001), involvement of seminal vesicles and/or lymph node metastasis (*P* < 0.001), and high level of HoxB13 expression (*P* < 0.001). In contrast, loss of PTEN expression (*P* < 0.001) was associated with longer BCR-free survival. Following multivariate analysis, DAC (*P* = 0.045), extension to the resection margin (*P* = 0.001) and high level of HoxB13 expression (*P* < 0.001) were associated with shorter BCR-free survival. In contrast, loss of PTEN expression (*P* = 0.049) was associated with longer BCR-free survival. The results of univariate and multivariate analyses are summarized in Table 3.

**Table 3:** Univariate and multivariate analysis of BCR-free survival in 178 prostate cancers.

Category	Variables	BCR-free survival			
		Univariate		Multivariate	
		HR (95% CI)	*P-*value	HR (95% CI)	*P-*value
Age (y)*	≤67	1		—	
	>67	1.132 (0.703–1.823)	0.610	—	—
Histologic subtype	AAC	1		1	
	DAC	4.127 (2.422–7.032)	<0.001	1.907 (1.015–3.585)	0.045
Gleason score	8	1		—	
	9–10	2.777 (1.721–4.482)	<0.001	—	—
Location	Unilateral	1		—	
	Bilateral	2.169 (1.070–4.396)	0.032	—	—
Tumor volume	≤5 cc	1		—	
	>5 cc	3.997 (2.447–6.529)	<0.001	—	—
EPE	Absent	1		1	
	Present	2.986 (1.814–4.914)	<0.001	0.975 (0.506–1.880)	0.940
Perineural invasion	Absent	1		—	
	Present	0.729 (0.315–1.687)	0.460	—	—
LVI	Absent	1		1	
	Present	2.707 (1.521–4.817)	0.001	0.882 (0.413–1.882)	0.745
RM extension	Absent	1		1	
	Present	2.789 (1.608–4.836)	<0.001	2.957 (1.546–5.655)	0.001
SVI and/or LNM	Absent	1		1	
	Present	3.308 (2.008–5.450)	<0.001	1.862 (0.977–3.547)	0.059
Pathologic T stage	T2	1		—	
	T3 and T4	3.098 (1.853–5.177)	<0.001	—	—
HoxA13 IHC	Low	1		—	
	High	0.939 (0.562–1.569)	0.810	—	—
HoxB13 IHC	Low	1		1	
	High	6.742 (3.721–12.217)	<0.001	4.293 (2.013–9.152)	<0.001
PTEN IHC	Loss	1		1	
	Intact	0.331 (0.182–0.603)	<0.001	0.531 (0.282–0.999)	0.049
ERG IHC	Negative	1		—	
	Positive	0.616 (0.303–1.251)	0.180	—	—

Abbreviations: DAC, ductal type adenocarcinoma; AAC, acinar type adenocarcinoma; EPE, extraprostatic extension; LVI, lymphovascular invasion; RM, resection margin; SVI, seminal vesicle involvement; LNM, lymph node metastasis; IHC, immunohistochemistry

*Median age of 178 patients was 67.0 y.

### Proposed diagnostic algorithm for DAC

**Figure 5:**
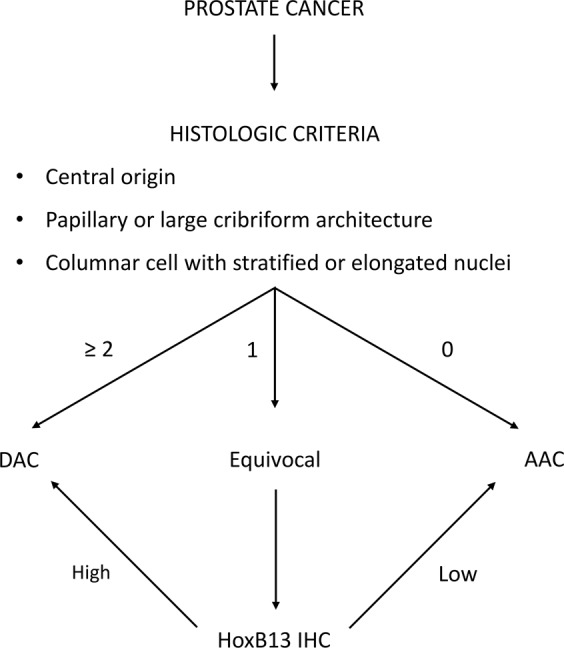
Proposed diagnostic algorithm for DAC. 0–2: number of satisfied criteria, +: HoxB13 positivity, −: HoxB13 negativity.

*Morphologic criteria* and *immunophenotypic criteria* showed strong correlation in the training set (Spearman’s correlation coefficient = 0.685, *P* < 0.001) and the test set (Spearman’s correlation coefficient = 0.657, *P* < 0.001), respectively. In addition, the *immunophenotypic criteria* showed superior interobserver agreement compared to *morphologic criteria*. Thus, HoxB13 immunohistochemistry (IHC) can be used as a diagnostic marker for DAC in cases with uncertain morphologic features. Based on these findings, we propose a new diagnostic algorithm for DAC (Fig. 5).

## Discussion

Histologic subtypes other than AAC represent less than 10% of all prostate cancer cases^21^. DAC is usually combined with AAC to yield mixed type adenocarcinomas. According to several studies, the proportion of cases classified as this mixed type adenocarcinoma varies from 0.13% to 12.7%^6,21–23^. Although less frequent, the biologic behavior of DAC is aggressive, exhibiting frequent EPE, involvement of seminal vesicles, extension to the surgical resection margins, presence of LVI, and BCR^4,24^. Moreover, DAC also metastasizes to unusual sites, such as the lung, liver, and brain^25^.

Despite the high mortality rate of DAC, it is difficult to detect this tumor type because of the frequent subnormal prostate-specific antigen (PSA) levels^26^. A study about the interobserver variability in the diagnosis of DAC was conducted and papillary architecture was proven to be the most important factor for the diagnosis of DAC^3^. However, despite several studies to elucidate the immunoprofile of DAC^9–11^, definitive diagnostic markers for DAC have not yet been identified.

In this study, we evaluated the morphological patterns of DAC and the differences in expression of HoxA13 and HoxB13 between DAC and AAC. HoxB13 was strongly expressed in Gleason pattern 4 DACs. In addition, HoxB13 was expressed in tumor cells that exhibited tubular structures or growth patterns equivalent to Gleason pattern 5 of AAC. These findings raise the possibility of variable Gleason patterns, including 3 and 5, for DACs. DACs with Gleason pattern 3 and 5 were observed in the vicinity of DAC nodules. DACs with tubular features (Gleason pattern 3) were more frequently identified than DACs with Gleason pattern 5; however, DACs with Gleason pattern 3 formed larger tubular glands than those of AAC and were composed of tall columnar amphophilic cells with pseudostratification. DACs having Gleason pattern 5 manifested as central comedo-type necrosis or infiltrative cord-like patterns mimicking invasive lobular carcinoma of the breast. These Gleason pattern 5 DACs were comparatively larger in size than AACs and intermingled with Gleason pattern 4 DACs. However, we did not identify any other variants with a Gleason pattern 5, such as a solid sheet-like growth corresponding to that of AAC. Further studies are necessary to investigate the various variants with Gleason pattern 3 and 5 in DACs.

No significant correlation was identified between HoxA13 expression and histologic subtype by *morphologic criteria*; however, the majority of DAC cases showed high level of HoxB13 expression and were associated with intact PTEN protein expression and ERG negativity, consistent with previous study results^10^. In a previous study, Morais *et al*. suggested the possibility of a clonal relationship between ductal and acinar components of mixed type adenocarcinomas^10^. In addition, the *HoxB13* gene regulates luminal differentiation of prostatic epithelium in animal models^13^. Thus, it is plausible to assume that the expression level of the *HoxB13* gene is associated with the development of DAC. Further studies are required to elucidate the relationship between HoxB13 expression and the development of DAC.

We identified a strong correlation between subgroups based on the *morphologic* and *immunophenotypic criteria* in the training set and the test set. In addition, interobserver agreement based on the *immunophenotypic criteria* was better than that based on the *morphologic criteria* in both cohorts. In addition to diagnostic reproducibility, the changes in diagnostic criteria affected prognostic classification of prostate cancer patients. Before the application of *morphologic criteria*, no significant differences in BCR-free survival were identified between AAC and DAC; however, after the application of *morphologic criteria*, DACs were found to have a shorter BCR-free survival than AACs. In addition, after application of the *immunophenotypic criteria*, the equivocal cases with high level of HoxB13 expression exhibited BCR-free survival similar to that of DACs. Thus, our findings suggest that *immunophenotypic criteria* could be useful to determine the histologic subtypes of equivocal cases.

Upon univariate and multivariate analysis, the high level of HoxB13 expression was identified as a significant factor for the prediction of BCR, which is the similar findings to those of the previous study^27^. Based on our IHC and survival analyses results, we conclude that HoxB13 can be used as a diagnostic marker for DAC. In addition, HoxB13 expression can also be used as a prognostic marker, regardless of histologic subtype. Despite these promising findings, this study has a limitation because it is performed on the single cohort from the single institute. Therefore, additional studies using larger and independent cohorts are necessary to validate these conclusions.

In summary, we investigated the morphological features of DAC and the expression of HoxB13 in 178 radical prostatectomy (RP) specimens. DACs showed various morphological features that lead to diagnostic difficulties; however, using HoxB13 expression analysis for *immunophenotypic criteria* combined with morphologic characteristics resulted in improved interobserver agreement and prognostic significance. Therefore, we suggest that when a final diagnosis remains equivocal, HoxB13 IHC can be an excellent ancillary measure to diagnose DAC.

## Materials and Methods

### Patient selection and clinical information

All 1460 consecutive RP specimens from 2008 to 2014 were selected from the archive of the Severance Hospital. Cases with neoadjuvant androgen deprivation therapy were excluded. To rule out the possibility of other conditions, such as HGPIN or IDC-P, that mimic DAC, dual IHC for high molecular weight cytokeratin and α-methylacyl-CoA racemase was performed. After evaluation of hematoxylin and eosin (H&E)-stained slides and dual IHC, 75 cases, including 15 mixed-type adenocarcinoma cases, were eventually selected as the DAC group. As a control group, 103 consecutive RP specimens diagnosed as AAC from 2008 to 2014 were included and matched with a corresponding Gleason score of ≥ 8. The entire cases were randomly assigned to 99 cases of training set and 79 cases of test set.

Several clinical factors, including age at the time of operation, follow-up level of PSA, and other follow-up data were obtained via medical record review. Cases with serum PSA > 0.2 ng/mL at least 6 weeks after surgery and a second confirmatory increase thereafter were considered to have BCR. BCR-free time was estimated from the date of the first curative surgery to the date of BCR or death without any type of relapse. This study was approved by the Institutional Review Board of the Severance Hospital (4-2018-0641) and informed consent were obtained from all patients. This study was performed in accordance with the Declaration of Helsinki.

### Histopathological evaluation

All cases were reviewed by three independent pathologists via evaluation of H&E-stained whole-section slides. Pathologic factors, including Gleason score (based on the 2014 International Society of Urological Pathology consensus)^28^, EPE, LVI, perineural invasion, extension to resection margin, seminal vesicle involvement, and pathologic stage based on the 8th American Joint Committee on Cancer criteria^29^ were acquired. Tumor volume was calculated by visual inspection method as previously described^30^.

For the diagnosis of DAC, we newly defined the following as diagnostic criteria for the ductal component: (1) topographical origin of central primary ducts close to urethral lumen, (2) true papillary and/or cribriform architecture more than three times larger than typical acini, (3) tall columnar epithelium that was at least three times longer than the height of the nuclei and stratified or elongated nuclei with prominent nucleoli. Cases that satisfied at least two of diagnostic criteria were classified as DAC. Equivocal cases that satisfied only one criterion were considered to be AAC.

### IHC and interpretation

The antibodies used for IHC on formalin-fixed paraffin-embedded tissue whole sections are shown in Supplementary Table S2. IHC was conducted with the Ventana Discovery XT automated stainer (Ventana Medical Systems, Tucson, AZ, USA) according the manufacturer’s protocol. Cell Conditioning 1 buffer (EDTA, pH 8.0, Ventana Medical Systems) was used for antigen retrieval.

Interpretation of IHC results was performed by a urologic pathologist. Cytoplasmic staining of HoxA13 and HoxB13 was considered non-specific, and only nuclear staining was evaluated. The results of HoxA13 and HoxB13 IHC were evaluated using a classification system based on the proportion and intensity of staining, as previously described^31^. The proportion category was assigned as follows: 1 = 0–4%, 2 = 5–19%, 3 = 20–39%, 4 = 40–59%, 5 = 60–79%, and 6 = 80–100%. Intensity category was assigned as follows: 0 = no staining, 1 = weak, 2 = intermediate, and 3 = strong. Quickscore was defined as the product of the proportion and intensity scores^31^. Quickscores ≥3 were considered to be high expression, and those <3 were regarded as low expression.

PTEN expression was evaluated by comparing staining between malignant glands and adjacent benign glands or stroma. As previously described, cases with markedly decreased or completely negative staining across entire tumor glands compared with the adjacent benign glands or stroma were considered to have loss of PTEN expression^10,32^. The other cases were considered to have intact PTEN expression. Cases with any tumor cells showing nuclear ERG expression were considered positive for expression.

### Evaluation of correlation and interobserver agreement

Samples were examined in a double-blind manner by two independent experienced pathologists (YA Cho [observer A] and JW Joo [observer B]). Both pathologists were also blinded to the results of the histologic evaluation, which was performed by other pathologists. Before training, interobserver agreement was evaluated on the training set. After that, two independent pathologists were trained via morphologic criteria for DAC and IHC slides of HoxB13 that were matched to the H&E-stained slides. The results of HoxB13 IHC performed by the urologic pathologist were blinded to the observers. And then, interobserver agreement was evaluated on the training set and the test set after each training.

### Statistical analysis

Data were analyzed using SPSS for Windows version 21.0 (SPSS Inc., Chicago, IL, USA). Student’s *t*-test and chi-square test were used for continuous and categorical variables, respectively. Kaplan-Meier survival curves and log-rank statistics were employed to evaluate time to tumor metastasis and time to survival. Multivariate regression analysis was performed using a Cox proportional hazards model. Statistical significance was assumed when *P* < 0.05.

Spearman’s correlation coefficient was used to evaluate correlation between two diagnostic criteria, and interobserver agreement was evaluated by calculating percentage agreement. Cohen’s kappa was used to compare the observed agreement and that expected by chance. Kappa values were categorized as previously described^33^.

## Supplementary information

Supplementary information.

## Data Availability

All data of this study are available from the corresponding author on reasonable request.
