# Supplementary material for: HoxB13 expression in ductal type adenocarcinoma of prostate: clinicopathologic characteristics and its utility as potential diagnostic marker
Source: Sci Rep. 2019 Dec 27;9:20205. doi: 10.1038/s41598-019-56657-8 (PMC6934792; doi:10.1038/s41598-019-56657-8)
Supplement: Supplementary file 1 — Supplementary information. [file 41598_2019_56657_MOESM1_ESM.docx]

**HoxB13 expression in ductal type adenocarcinoma of prostate: clinicopathologic characteristics and its utility as potential diagnostic marker**

Cheol Keun Park^1,2^, Su-Jin Shin^3^, Yoon Ah Cho^1,4^, Jin Woo Joo^1^, and Nam Hoon Cho^1,*^

^1^Department of Pathology, Severance Hospital, Yonsei University College of Medicine, Seoul, Republic of Korea

^2^Department of Pathology, Armed Forces Capital Hospital, Seongnam, Republic of Korea

^3^Department of Pathology, Hanyang University College of Medicine, Seoul, Republic of Korea

^4^Department of Pathology and Translational genomics, Samsung medical center, Seoul, Republic of Korea

*Correspondence and requests for materials should be addressed to N.H.C. (cho1988@yuhs.ac)

| **Supplementary Table S1.** Clinicopathological characteristics of 178 prostate cancers HoxA13 expression status in training set and test set | | | | | | | | | | | | | |
| --- | --- | --- | --- | --- | --- | --- | --- | --- | --- | --- | --- | --- | --- |
| Category | Variables | Training set | | | | | | Test set | | | | | |
|  |  | No. of cases  (*n*=99) | High (%) | | Low (%) | | *P-*value | No. of cases  (*n*=79) | High (%) | | Low (%) | | *P-*value |
|  |  |  | (*n*=60) | | (*n*=39) | |  |  | (*n*=46) | | (*n*=33) | |  |
| Age (y) |  |  | 67.8 ± 7.54 | | 64.9 ± 7.76 | | 0.069 |  | 66.6 ± 6.58 | | 64.4 ± 7.60 | | 0.172 |
| Pre-operative PSA  (ng/mL) |  |  | 13.1 ± 15.8 | | 10.2 ± 6.37 | | 0.281 |  | 10.2 ± 7.19 | | 10.6 ± 7.37 | | 0.800 |
| Histologic subtype | AAC | 63 | 39 | (65.0) | 24 | (61.5) | 0.726 | 47 | 28 | (60.9) | 19 | (57.6) | 0.769 |
|  | DAC | 36 | 21 | (35.0) | 15 | (38.5) |  | 32 | 18 | (39.1) | 14 | (42.4) |  |
| Gleason score | 8 | 69 | 41 | (68.3) | 28 | (71.8) | 0.714 | 55 | 35 | (76.1) | 20 | (60.6) | 0.140 |
|  | 9-10 | 30 | 19 | (31.7) | 11 | (28.2) |  | 24 | 11 | (23.9) | 13 | (39.4) |  |
| Location | Unilateral | 23 | 16 | (26.7) | 7 | (17.9) | 0.316 | 14 | 8 | (17.4) | 6 | (18.2) | 0.928 |
|  | Bilateral | 76 | 44 | (73.3) | 32 | (82.1) |  | 65 | 38 | (82.6) | 27 | (81.8) |  |
| Tumor volume | ≤5 cc | 74 | 41 | (68.3) | 33 | (84.6) | 0.068 | 60 | 35 | (76.1) | 25 | (75.8) | 0.973 |
|  | >5 cc | 25 | 19 | (31.7) | 6 | (15.4) |  | 19 | 11 | (23.9) | 6 | (24.2) |  |
| EPE | Absent | 52 | 25 | (41.7) | 27 | (69.2) | 0.008 | 47 | 28 | (60.9) | 19 | (57.6) | 0.769 |
|  | Present | 47 | 35 | (58.3) | 12 | (30.8) |  | 32 | 18 | (39.1) | 14 | (42.4) |  |
| PNI | Absent | 7 | 4 | (6.7) | 3 | (7.7) | >0.999 | 5 | 3 | (6.5) | 2 | (6.1) | >0.999 |
|  | Present | 92 | 56 | (93.3) | 36 | (92.3) |  | 74 | 43 | (93.5) | 31 | (93.9) |  |
| LVI | Absent | 88 | 51 | (85.0) | 37 | (94.9) | 0.192 | 67 | 38 | (82.6) | 29 | (87.9) | 0.520 |
|  | Present | 11 | 9 | (15.0) | 2 | (5.1) |  | 12 | 8 | (17.4) | 4 | (12.1) |  |
| RM extension | Absent | 34 | 20 | (33.3) | 14 | (35.9) | 0.793 | 42 | 26 | (56.5) | 16 | (48.5) | 0.480 |
|  | Present | 65 | 40 | (66.7) | 25 | (64.1) |  | 37 | 20 | (43.5) | 17 | (51.5) |  |
| SV involvement | Absent | 82 | 49 | (81.7) | 33 | (84.6) | 0.704 | 69 | 41 | (89.1) | 28 | (84.8) | 0.734 |
|  | Present | 17 | 11 | (18.3) | 6 | (15.4) |  | 10 | 5 | (10.9) | 5 | (15.2) |  |
| Pathologic T stage | T2 | 46 | 22 | (36.7) | 24 | (61.5) | 0.015 | 47 | 28 | (60.9) | 19 | (57.6) | 0.769 |
|  | T3 and T4 | 53 | 38 | (63.3) | 15 | (38.5) |  | 32 | 18 | (39.1) | 14 | (42.4) |  |
| Pathologic N stage* | N0 | 68 | 38 | (90.5) | 30 | (100.0) | 0.135 | 40 | 22 | (100.0) | 18 | (94.7) | 0.463 |
|  | N1 | 4 | 4 | (9.5) |  |  |  | 1 |  |  | 1 | (5.3) |  |
| PTEN IHC | Intact | 55 | 31 | (51.7) | 24 | (61.5) | 0.334 | 46 | 29 | (63.0) | 17 | (51.5) | 0.306 |
|  | Loss | 44 | 29 | (48.3) | 15 | (38.5) |  | 33 | 17 | (37.0) | 16 | (48.5) |  |
| ERG IHC | Negative | 83 | 51 | (85.0) | 32 | (82.1) | 0.697 | 60 | 36 | (78.3) | 24 | (72.7) | 0.570 |
|  | Positive | 16 | 9 | (15.0) | 7 | (17.9) |  | 19 | 10 | (21.7) | 9 | (27.3) |  |
| BCR | Absent | 57 | 37 | (61.7) | 20 | (51.3) | 0.307 | 61 | 34 | (73.9) | 27 | (81.8) | 0.409 |
|  | Present | 42 | 23 | (38.3) | 19 | (48.7) |  | 18 | 12 | (26.1) | 6 | (18.2) |  |
| Abbreviations: DAC, ductal type adenocarcinoma; AAC, acinar type adenocarcinoma; PSA, prostate-specific antigen; EPE, extraprostatic extension; PNI, perineural invasion; LVI, lymphovascular invasion; RM, resection margin; SV, seminal vesicle; IHC, immunohistochemistry; BCR, biochemical recurrence  *Evaluated in 113 prostatectomy specimens. | | | | | | | | | | | | | |

| **Supplementary Table S2.** Antibodies used for immunohistochemistry | | | |
| --- | --- | --- | --- |
| Antibody | Source | Clone | Dilution |
| HMW-CK | DAKO, Glostrup, Denmark | 34betaE12 | 1:50 |
| AMACR | DAKO, Glostrup, Denmark | 13H4 | 1:100 |
| HoxA13 | Abcam, Cambridge, UK | Polyclonal | 1:50 |
| HoxB13 | Abcam, Cambridge, UK | Polyclonal | 1:300 |
| PTEN | Cell Signaling Technology, Danvers, MA, USA | D4.3 | 1:100 |
| ERG | Abcam, Cambridge, UK | EPR3864 | 1:200 |
| Abbreviations: HMW-CK, High molecular weight cytokeratin; AMACR, α-methylacyl-CoA racemase; HoxA13, Homeobox A13; HoxB13, Homeobox B13; PTEN, phosphatase and tensin homolog; ERG, ETS-related gene | | | |

**
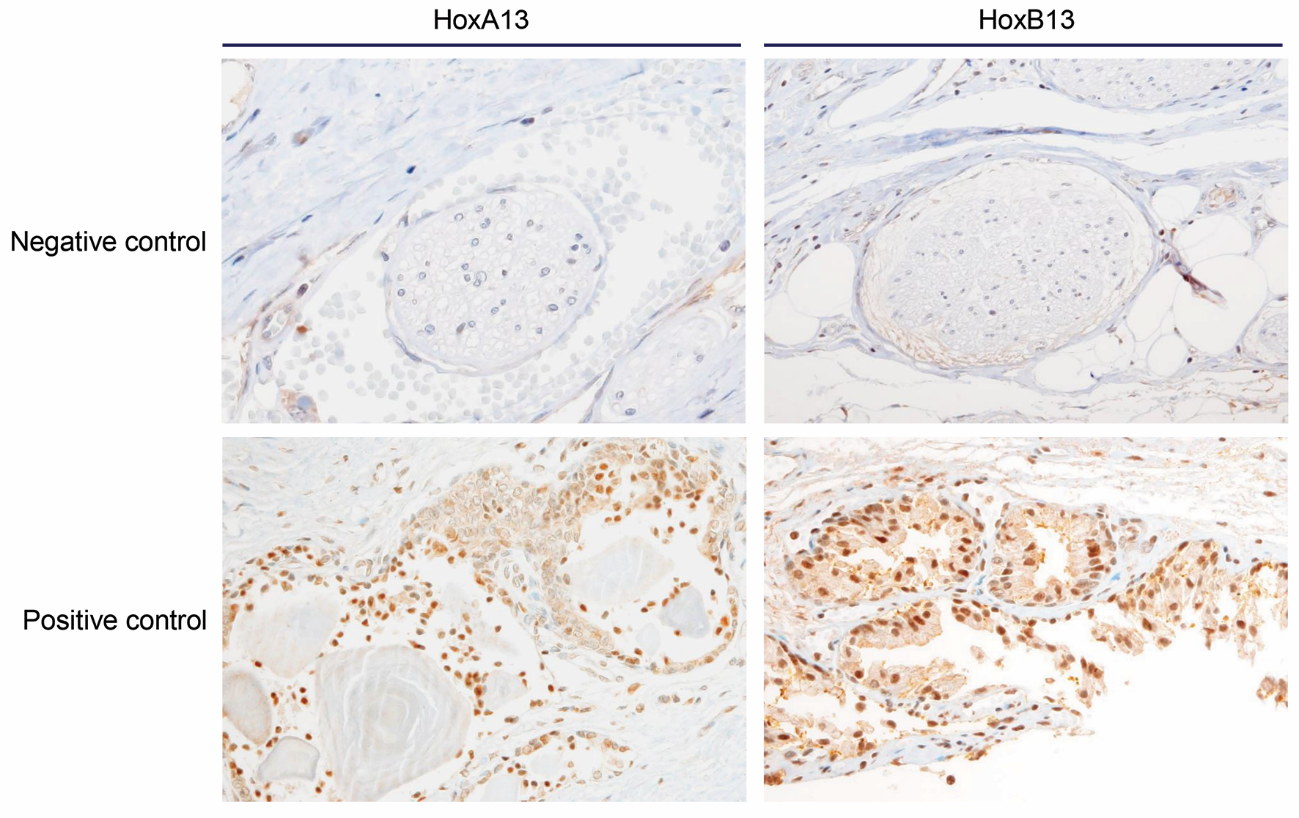
**

**Supplementary Figure 1. The validation results of HoxA13 and HoxB13 antibody.** Neither HoxA13 nor HoxB13 is expressed in the negative control (nerve bundles or periprostatic adipose tissue). Both HoxA13 and HoxB13 are expressed in the nuclei of normal prostatic epithelium.


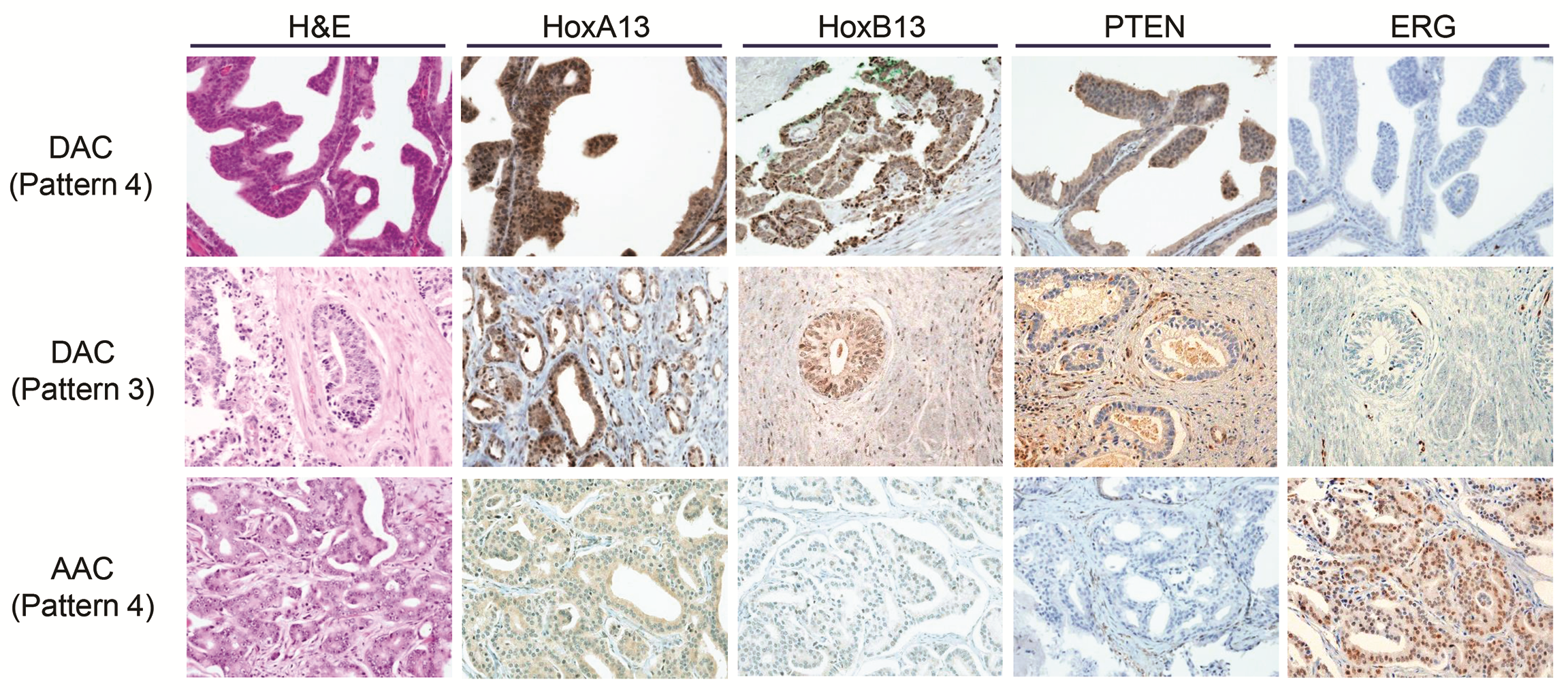


**Supplementary Figure 2. Representative immunoprofiles of DAC and AAC.** In DACs, the tumor compartment that corresponds to Gleason pattern 3 and 4 showed similar immunoprofiles: high expression of HoxA13 and HoxB13, intact PTEN, and ERG negativity. In contrast, AACs showed different immunoprofiles than those of DACs: low expression of HoxA13, HoxB13 negativity, loss of PTEN expression, and ERG positivity.
